# Supplementary material for: Naphthalene-Based Polymers as Catalytic Supports for Suzuki Cross-Coupling
Source: Molecules. 2023 Jun 23;28(13):4938. doi: 10.3390/molecules28134938 (PMC10343485; doi:10.3390/molecules28134938)
Supplement: Supplementary file 1 [file molecules-28-04938-s001.zip › molecules-2402981-supplementary.pdf]

molecules

MDPI

Supplementary Materials

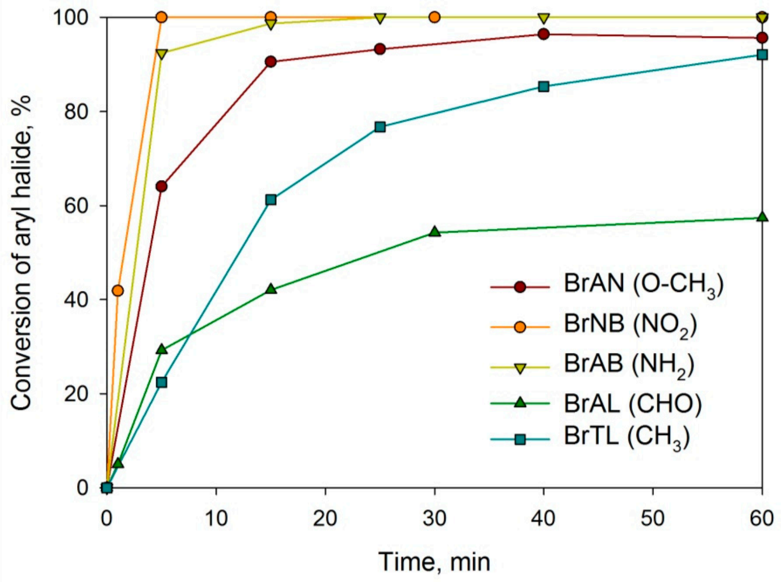

Figure S1. Scope of aryl halides for 1%-Pd/SNA120 (1 mmol of aryl halide, 1.5 mmol of PBA, 2.0 mmol of NaOH, 60°C, 900 rpm, 0.02 g of the catalyst).

Table S1. Influence of aryl halide nature on the Suzuki cross-coupling in the presence of the catalyst 1%-Pd/SNA120.

Aryl Halide	Conversion of BrAN, %	Yield of Cross-Coupling Product, %	R_0, $mol_{BrAN}/(mol_{Pd}*min)$
BrAN	95.6	91.6	64.0
BrNB	100	>99	209.2
BrAB	100	56.0	92.4
BrAL	54.4	48.3	25.1
BrTL	92.1	81.5	22.4

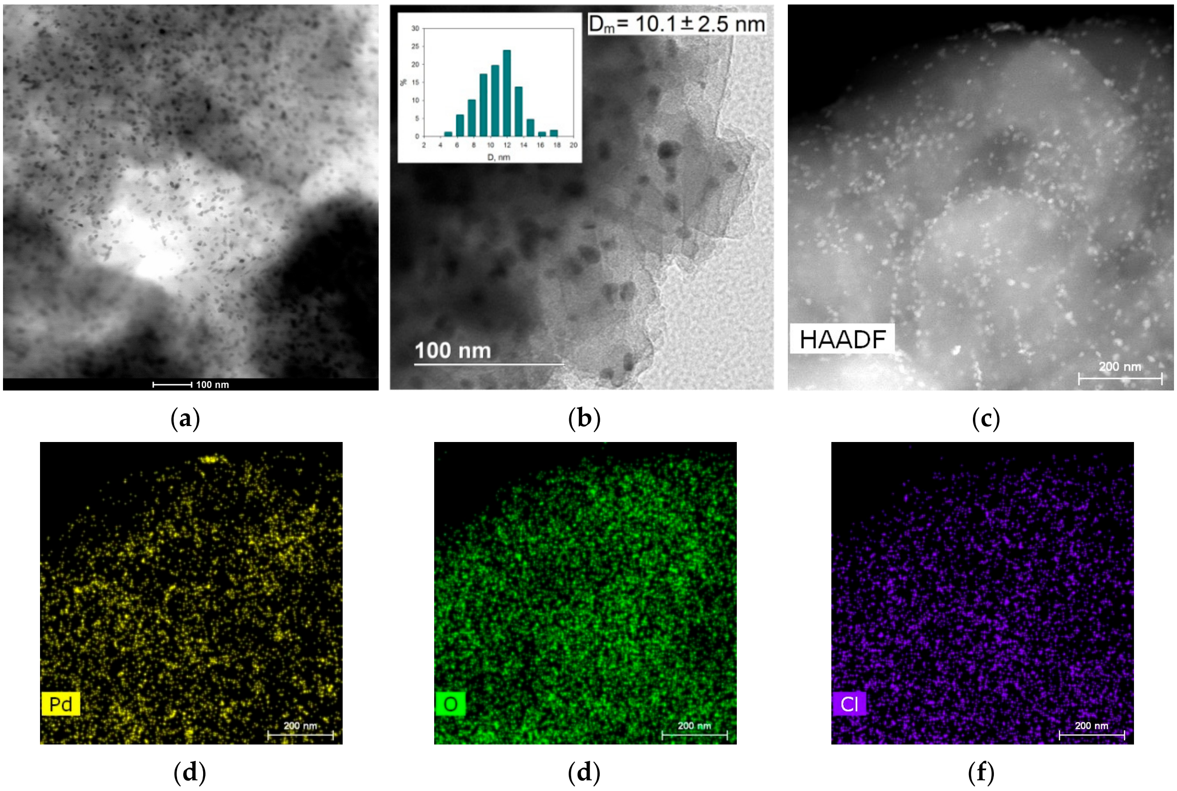

Figure S2. Bright-field STEM (scale 100 nm) (**a**, **b**) and HAADF STEM (scale 200 nm) (**c**) images of the sample 1%-Pd/NL60 taken after the second use in Suzuki reaction and EDX mapping of Pd (**d**), O (**e**), and Cl (**f**).

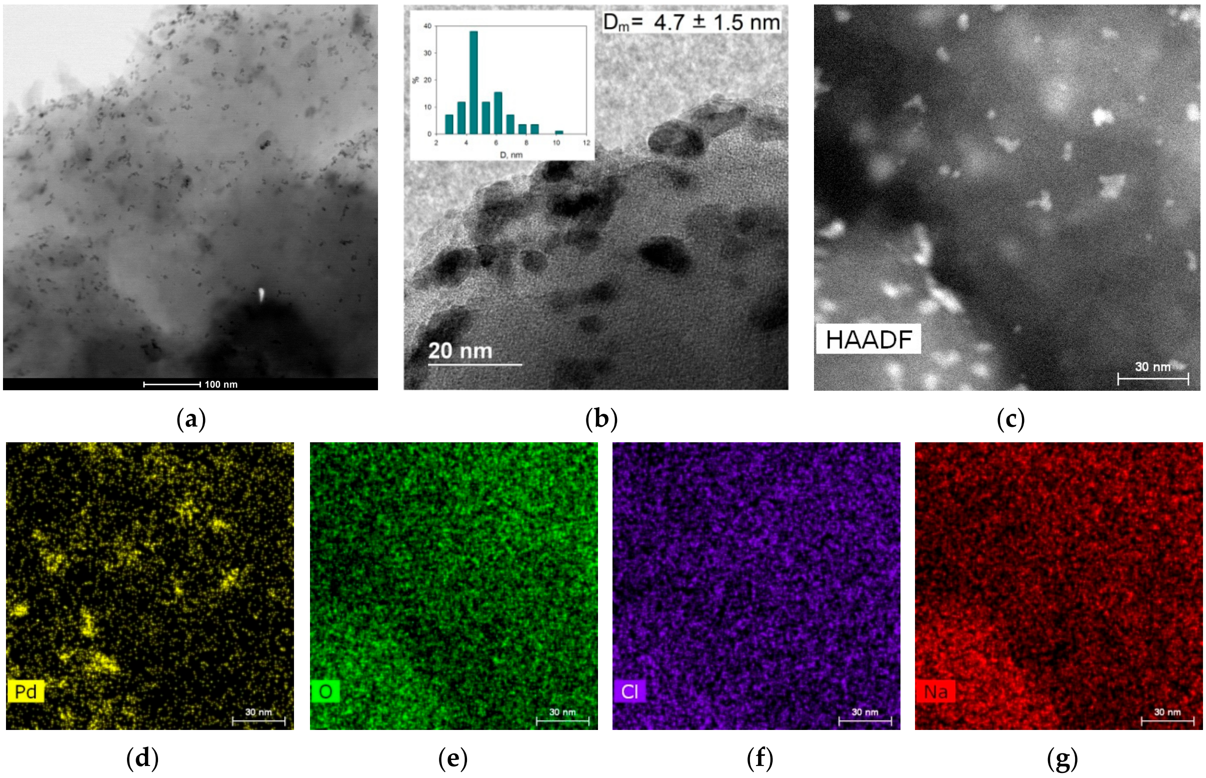

Figure S3. Bright-field STEM (scales 100 and 20 nm) (**a**, **b**) and HAADF STEM (scale 30 nm) (**c**) images of the sample 1%-Pd/NNA120 taken after the second use in Suzuki reaction and EDX mapping of Pd (**d**), O (**e**), Cl (**f**), and Na (**g**).

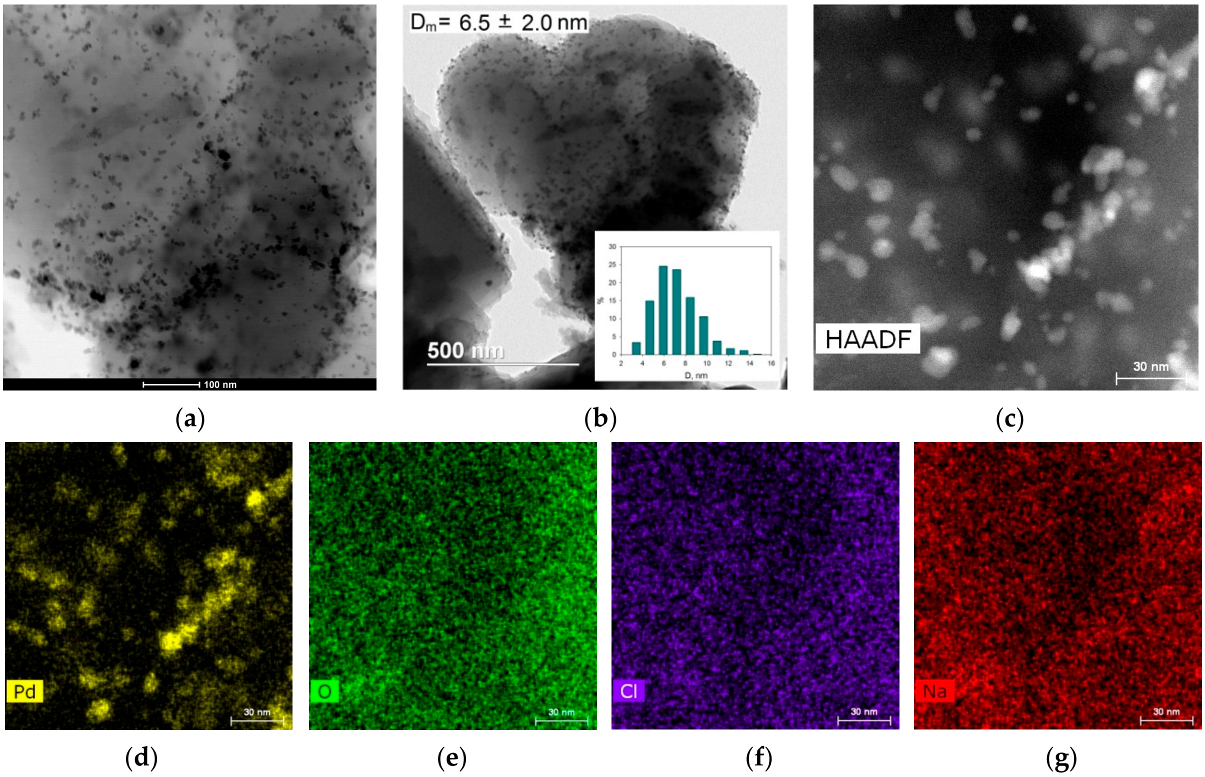

Figure S4. Bright-field STEM (scales 100 and 500 nm) (**a**, **b**) and HAADF STEM (scale 30 nm) (**c**) images of the sample 1%-Pd/SNA120 taken after the second use in Suzuki reaction and EDX mapping of Pd (**d**), O (**e**), Cl (**f**), and Na (**g**).

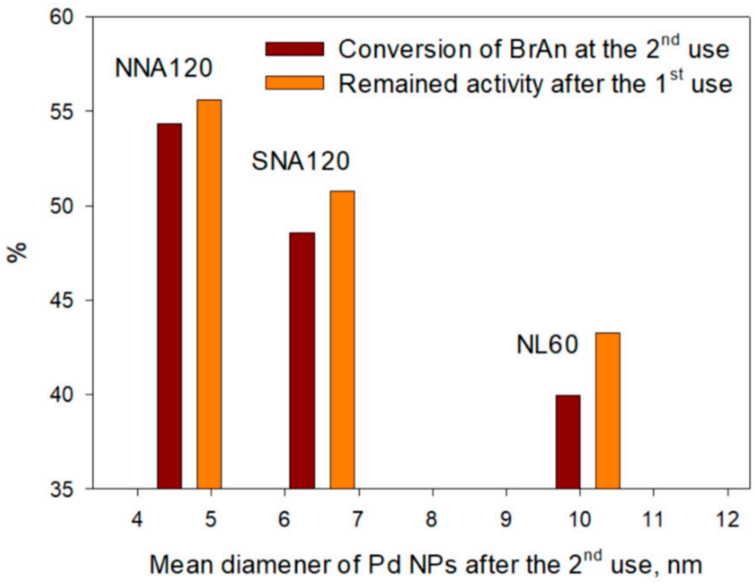

Figure S5. Correlation between the activity and stability in the second run on the sizes of Pd NPs formed during the second run in the initial (unreduced) samples (1 mmol of BrAN, 1.5 mmol of PBA, 2.0 mmol of NaOH, 60°C, 900 rpm, 0.02 g of the catalyst).

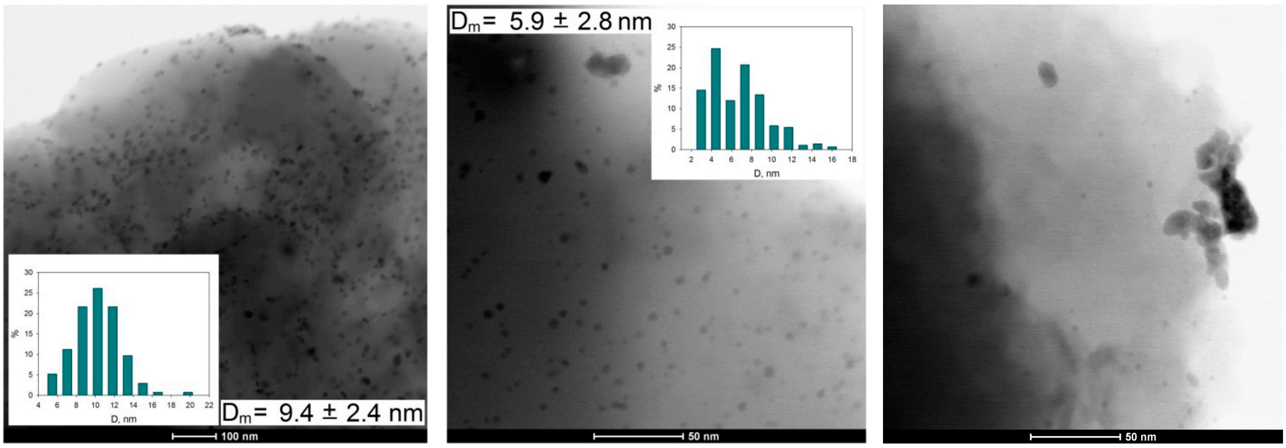

(**a**) (**b**) (**c**)

Figure S6. Bright-field STEM images of 1%-Pd/NL60-R taken after the second use in Suzuki reaction (scale 100 nm) (**a**), 1%-Pd/SNA120-R (**b**), and 1%-Pd/NNA120-R (**c**) both taken after the third use in Suzuki reaction (scale 50 nm).

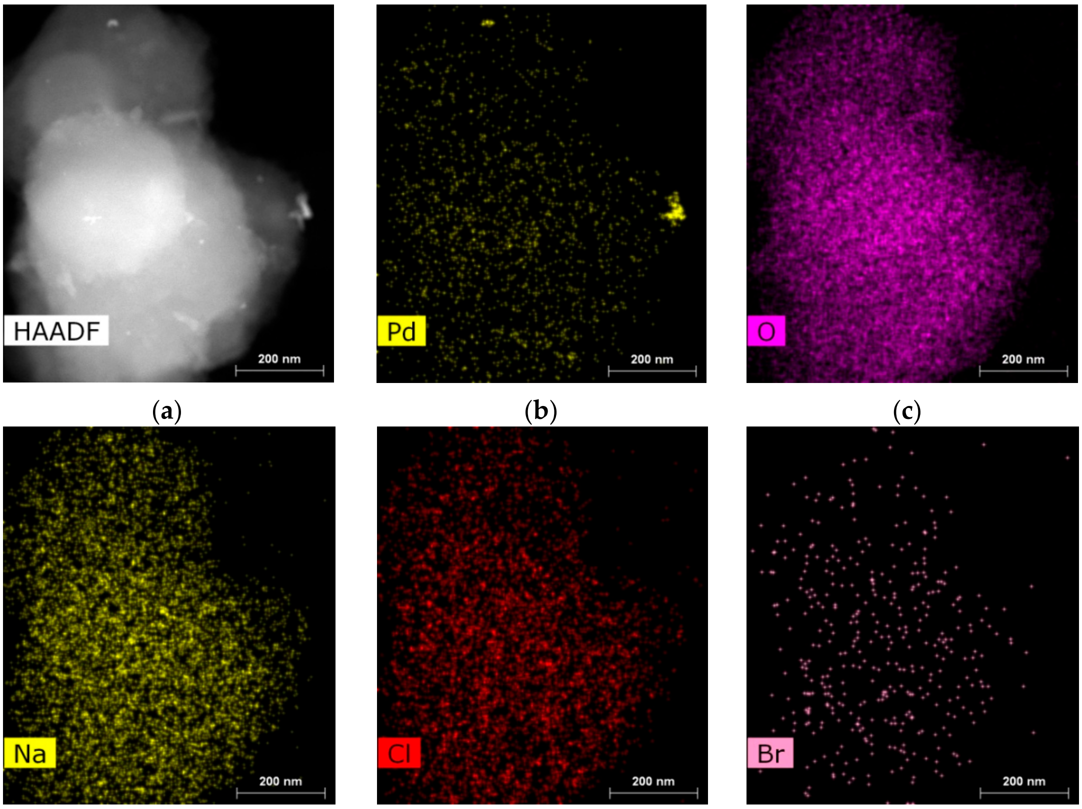

(**d**) (**e**) (**f**)

Figure S7. HAADF STEM image (scale 200 nm) (**a**) of the sample 1%-Pd/NNA120-R taken after the third use in Suzuki reaction and EDX mapping of Pd (**b**), O (**c**), Na (**d**), Cl (**e**), and Br (**f**).

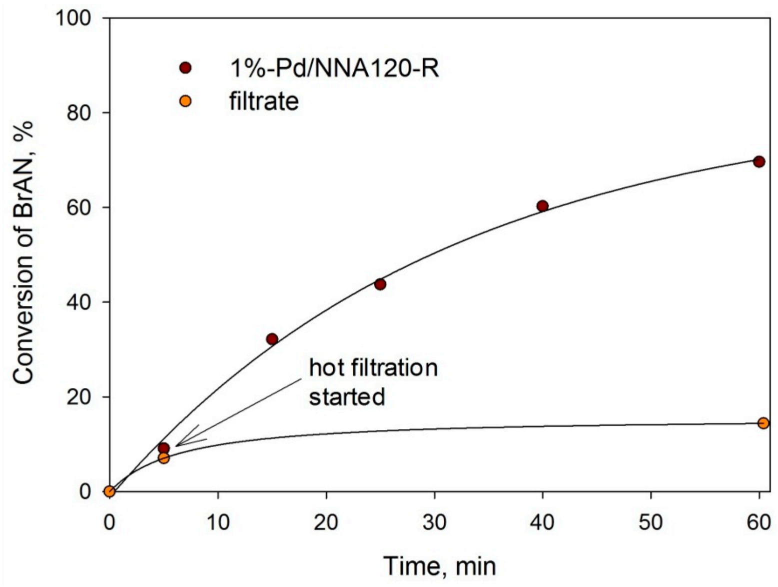

Figure S8. Hot-filtration test for 1%-Pd/NNA120-R (1 mmol of BrAN, 1.5 mmol of PBA, 2.0 mmol of NaOH, 60°C, 900 rpm, 0.02 g of the catalyst).
